# Role of Intraoperative Sentinel Lymph Node Mapping in the Management of Carcinoma of the Esophagus

**DOI:** 10.4103/1319-3767.65186

**Published:** 2010-07

**Authors:** Mohammad A. Bhat, Zahoor A. Naikoo, Tufail A. Dass, Riyaz A. Lone, Abdul M. Dar

**Affiliations:** Department of Cardiovascular and Thoracic Surgery, Sheri-Kashmir Institute of Medical Sciences, Srinagar -190 011, Kashmir, India

**Keywords:** Anastomosis, esophageal cancer, esophagus, surgery

## Abstract

**Background/Aim:**

Precise evaluation of lymph node status is one of the most important factors in determining clinical outcome in treating gastro-intestinal (GI) cancer. Sentinel lymph node (SLN) mapping clearly has become highly feasible and accurate in staging GI cancer. This study aims to investigate the feasibility and accuracy of detection of SLN using methylene blue dye in patients with carcinoma of the esophagus and assess its potential role in determining the rational extent of lymphadenectomy in esophageal cancer surgery.

**Materials and Methods:**

Thirty-two patients of esophageal cancer diagnosed on endoscopic biopsy were enrolled in this prospective study. After laparotomy, patent methylene blue was injected into the subserosal layer adjacent to the tumor. SLNs were defined as blue stained nodes within a period of 5 min. Standard radical esophagogastrectomy with lymphadenectomy was performed in all the patients. All the resected nodes were examined postoperatively by routine hematoxylin and eosin stain for elucidating the presence of metastasis, and the negative SLNs were examined further with cytokeratin immunohistochemical staining.

**Results:**

SLNs were detected in 26 (81.25%) patients out of 32 patients who were studied. The number of SLNs ranged from 1 to 4 with a mean value of 1.7 per case. The SLNs of esophageal cancer were only found in N1 area in 21 (80.77%) cases, and in N2 or N3 area in only 19.33%. The overall accuracy of the procedure was 75% in predicting nodal metastasis. SLN had a sensitivity of 85.71% in mid esophageal tumors and 93.33% in lower esophageal tumors. The SLN biopsy had sensitivity of 87.5% in the case of squamous cell carcinoma and 92.86% in the cases of adenocarcinoma of the esophagus. The accuracy of the procedure for squamous cell carcinoma and adenocarcinoma was 60% and 76.47%, respectively.

**Conclusion:**

SLN mapping is an accurate diagnostic procedure for detecting lymph node metastasis in patients with esophageal cancer and may indicate rational extent of lymphadenectomy in these patients. SLN mapping provides “right nodes” to the pathologists for detailed analysis and appropriate staging, thereby helping in individualizing the multi-modal treatment for esophageal cancer.

Regional lymph node involvement is the most important prognostic indicator in patients with solid tumors and also holds true for carcinoma of esophagus. The tumor status of regional lymph nodes is crucial for staging gastrointestinal neoplasm.[1,2] Sentinel node (SLN) is the first lymph node draining the lymphatic basin from the primary tumor. Cabañas used lymphangiography for the visualization of the SLN in patients with carcinoma of penis.[3] After Morton *et al* demonstrated the clinical significance of SLN concept in melanoma, it has attracted vast attention in surgical oncology.[4] Though the validity of this concept has been investigated in breast, gastric and colorectal cancers using radio-active tracers, the application of this concept to gastrointestinal cancers, which have multidirectional complex lymphatic flow, is controversial. The clinical significance of SLN concept differs in various solid tumors depending on anatomical location and histological behavior of cancer cells. Extended lymphadenectomy is not always beneficial because of increased morbidity associated with it. Besides it is difficult to identify the patients who would be potentially curable with this approach, even if the patients are preoperatively evaluated with computerized tomography (CT), magnetic resonance imaging (MRI), and endoscopic ultrasonograhy (EUS). We explored the feasibility and significance of SLN biopsy in patients with esophageal cancer using intra-operative methylene blue dye.

## MATERIALS AND METHODS

This prospective study was conducted in a tertiary care institution (Sheri Kashmir Institute of Medical Sciences, Srinager, India) from October 2004 to October 2007. Thirty-two patients of esophageal cancer diagnosed on endoscopic biopsy were enrolled in this study. The study was approved by the Ethical Committee of the Institute. Informed consent was taken from all the patients before surgical intervention. The mean age of the patients was 52.5 years. The sex ratio showed a clear male preponderance, with male to female ratio of 5:3. Patients with clinical or radiological evidence of metastasis, T4 tumors, and concomitant co-morbid conditions, such as chronic obstructive pulmonary disease (with VC <1L, FEVI <50%); chronic renal failure (serum creatinine >3mgms/dl), were excluded from the study. Besides, patients who had undergone neoadjuvent therapy before surgery were excluded from the study. All patients were operated under general anesthesia. The patients with mid-esophageal lesions underwent thoracotomy first, and the patients having esophagogastric lesions underwent laparotomy first. Transhiatal esophagogastrectomy was only performed in those patients of lower esophageal neoplasia, where the residual stomach distal to the growth, reached the cervical region without tension.

Intraoperative lymphatic mapping was performed by injecting 0.5 ml of 1% methylene blue at four points at the periphery of the tumor (total 2 ml), before mobilization of the tumor was started. After visualizing the afferent channels, the lymphatic channels were dissected to identify the blue stained lymph nodes. All the lymph nodes that got stained within a period of 5 min were marked.

**Figure 1:**
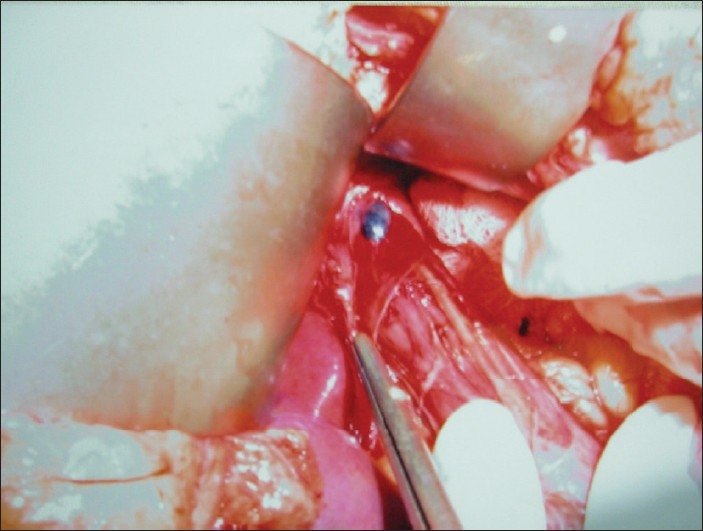
Intraoperative photograph showing a sentinel lymph node being dissected

**Figure 2:**
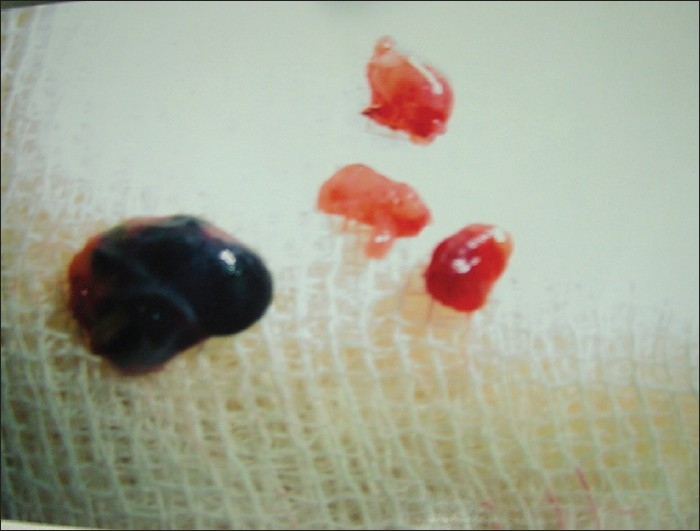
Photograph showing lymph nodes not stained with methylene blue and the sentinel lymph node

All the marked lymph nodes (blue nodes) were removed from the surgical specimen and the distance of lymph nodes from the primary tumor recorded [Figure 1]. If no lymph node stained within a period of 5 min, the patient was declared as SLN negative. All the positive nodes were sent for histopathological examination [Figure 2]. After that, en-bloc resection of the neoplasm was performed in a standard fashion.

The extent of lymphadenectomy in the upper abdominal compartment and lower posterior mediastinum was identical for all surgical approaches and comprised a suprapancreatic lymphadenectomy, including all lymph nodes along the common hepatic artery, celiac axis, and splenic artery toward the splenic hilum. The left gastric artery was always transected at its origin and remained with the specimen. Also included were all lymph nodes along the proximal two-thirds of the lesser gastric curvature and the gastric fundus, left and right paracardiac nodes, distal paraesophageal nodes, and nodes in the lower posterior mediastinum up to the tracheal bifurcation. With the transhiatal approach, this aspect was achieved after a wide anterior splitting of the diaphragmatic hiatus and transhiatal exposure of the lower posterior mediastinum.[5]

Patients with an abdomino-right-thoracic approach had an additional formal extended mediastinal lymphadenectomy comprising all nodes at the tracheal bifurcation along the left and right main stem bronchi, the upper mediastinal compartment, and along the left recurrent nerve. A systematic cervical lymphadenectomy was not performed routinely. The T-stage, total number of nodes and number of positive nodes were then recorded. Histopathological examination of blue stained (SLN) as well as un-stained nodes was performed using hematoxylin and eosin staining and the latter were examined further with cytokeratin immunohistochemical staining to rule out the presence of metastases.

### Statistical analysis

The results of SLN were quantified using the following definitions. Accuracy: (true positive+true negative)/(total patients), sensitivity (true positive)/(true positive+false negative), and negative predictive value: (true negative)/(true negative+false negative). *P* values that were two sided at 0.05 were considered statistically significant. Statistical package for social sciences (*SPSS, version* 10.0; Chicago, IL, USA) was used for data analysis.

## RESULTS

Out of 146 patients of carcinoma of the esophagus who were admitted for surgery over a period of 2 years, only 32 patients were randomly selected for sentinel lymph node mapping using methylene blue dye. Of the 32 patients studied, 5 (46.88%) had squamous cell carcinoma of esophagus and 17 (53.12%) had adenocarcinoma of esophagus. In 20 patients (62.5%), the tumors were located in the esophagogastric junction or lower esophagus and 12 (37.5%) had tumors in the mid-esophageal area [Table 1]. Majority of patients (71.88%) had pathologically advanced T_3_ tumors and only 28.12% patients had T_1_ or T_2_ tumors. The SLNs were only found in N1 area in 21 (80.77%) cases, and N2 or N3 area in only 5 patients (19.23%).

**Table 1:** Distribution of patients according to histopathological status of sentinel lymph nodes

Site of tumor	Type of tumor	SLN positive				SLN negative				Total No. of patients
		With metastases	Without metastases	Total	no.	With metastases	Without metastases	Total	No.	
Mid esophageal	Squamous cell carcinoma	4	3	7	9	1	1	2	3	12
	Adenocarcinoma	2	-	2		-	1	1		
Lower esophageal	Squamous cell carcinoma	3	1	4	17	-	2	2	3	20
	Adenocarcinoma	11	2	13		1	-	1		
Total No. of patients		20	6	26		2*	4	6		32

The SLN mapping appeared to be more sensitive in lower esophageal tumors (93.33%) than mid-esophageal lesions (85.71%), but this finding was not statistically significant (*P*>0.05). Detection was marginally better in adenocarcinoma of esophagus at 88.24% (15/17) compared with 73.34% (11/15) for squamous cell type [Table 1]. Average number of SLN identified per patient was 1.7 (range 1-3). Out of the 20 patients, in whom one or more SLNs were identified, the sentinel node was usually located within 5 cm. of the primary tumor in 80.76% of patients. In lower esophageal tumors, the SLN were located either in the periesophageal area or along the lesser curvature of the stomach. In one patient who had lower esophageal cancer, the SLN was located above the hiatus in mediastinum (at a distance of 6 cm) and in another case it was located along the lesser curve of stomach at approximately 10 cm from the primary lesion. In mid-esophageal tumors, out of the nine SLN positive cases, one of the sentinel nodes was found 8 cm from the tumor at the esophageal hiatus. The SLNs of esophageal cancer were only found in N1 area in 21 (80.77%) cases, and in N2 or N3 in only 19.33% cases. Among the 26 SLN positive patients, only 20(76.92%) showed metastases in the lymph nodes on histological examination. Of these 20 patients, 1 patient had metastases in non-stained nodes, whereas sentinel nodes were free of metastases [Table 1]. Out of the 20 patients in whom SLN had metastases, the SLN was the only site of metastases in 8 patients, whereas in remaining patients metastases were present in SLNs as well as non-stained nodes. Among the five patients in whom SLN could not be detected (SLN negative), only one patient was proven to have metastasis on histology. Of the two false negative cases, one was squamous cell carcinoma (mid esophageal) and the other adenocarcinoma (lower esophagus) type. Out of these two false negative cases, one had SLN at a distance of 6 cm from the primary lower esophageal tumor with metastases present in one of the paraesophageal lymph nodes.

The frequency of metastases in blue stained SLN (76.92%) was higher than that found in non-stained (SLN negative) nodes (23.08%) and was statistically significant (*P* < 0.05).

## DISCUSSION

Optimal management of esophageal cancer strategies include patient selection, accurate staging and risk assessment, selection of an appropriate surgical approach, and the use of multimodality treatment in the management of these patients. In addition, other factors such as hospital and surgeon volume are important in reducing the risks of esophagectomy.[6] Esophageal cancer is a particularly aggressive gastrointestinal malignancy because of high incidence and widespread distribution of lymph node metastasis. Kashmir has a high incidence of esophageal cancer, similar to the “Asian esophageal cancer belt.”[7] In esophageal cancer, selective removal of involved lymph nodes could improve survival and limit complications from extended lymphadenectomy.[8]

In our study, 15 (46.87%) patients had squamous cell carcinoma of esophagus and 17(53.12%) patients had adenocarcinoma of esophagus. This distribution is in contrast to the predominance of squamous cell carcinoma of esophagus reported by Law *et al*. in Asian population.[9] Out of the 32 patients, 28.12% had pT1 or pT2 tumor, whereas the remaining 71.88% patients had pT3 tumor. Kitagawa *et al*. conducted their study on T1 and T2 gastric cancer patients only,[10] and Song *et al*. had only 8 cases of advanced stage III disease out of 27 patients in their study.[11] The higher tumor stage in our study population is due to late presentation of the patients to the hospital. We considered only those lymph nodes as SLN positive that stained within a period of 5 min and any node which stained after that as SLN negative as recommended by Song *et al*[9] and Kitagawa *et al*.[12] since including the lymph nodes that stain beyond 5 min period increases the false positive rate steeply.

The studies that were conducted for evaluating the concept of SLN in esophagus have been mostly performed by using radioisotopes.[8,13] The dye method for identifying the SLNs has been used only in cases of gastric and colorectal carcinomas in the gastrointestinal tract. The overall detection rate of SLN in our study was 81.25% which is in agreement with various authors using radioisotopes and dye methods for SLN mapping[10,11,14] but is significantly higher than 65% reported by Guiliano *et al*.[15] Two patients with metastatic deposits in non-stained nodes were missed by the SLN mapping technique, probably due to advanced stage of the disease. In the advanced tumor stage, the lymphatics get blocked by the cancer cell permeation and emboli; the dye does not reach the SLN at all, leading to non-detection of SLNs.[16,17] The detection of SLN in our study was higher in lower esophageal tumors (85%) than in mid-esophageal tumors (75%), but it was not statistically significant (*P* value >0.05). The detection rate of sentinel nodes in adenocarcinoma of esophagus was marginally better than squamous cell carcinoma of esophagus (88.24% vs. 73.34%). Average number of SLNs detected was 1.7 nodes per patient (range 1-3 per patient) which is comparable to those reported in earlier series in carcinoma breast and melanoma. However, many authors have reported higher sentinel node detection by using dye or radioltracer methods (3.6-4.0 sentinel nodes /patient).[18–20] The lower number of sentinel nodes in our study could be explained by the fact that we labeled only those nodes as SLNs, which stained blue within 5 min of dye injection into the peritumoral area and tagged them for histopathological examination. Same technique was utilized by Song *et al*. in gastric cancer using isosulfan blue dye and reported detection of 2.7 nodes per patient which is comparable to our results.[11] Kitagawa *et al* have also recommended 2 to 5 min time interval for study of SLNs after dye injection.[12] The window of observation in the dye method is narrow, as the dye travels quickly and stains additional nodes beyond the sentinel nodes.[21] This is the reason for discrepancy in the results of earlier publications, wherein some authors have reported a higher number of sentinel node detection using a delayed identification technique. The average size of sentinel nodes in our study was 6.2 mm (range 3-20mm), which is in conformity with the published reports.[22,23]

The sentinel node was located within 5 cm of the primary tumor in 80.76% patients, but SLN was not the node nearest to the tumor in 42.31% of the patients (11/26). The same observations were made by Kitagawa *et al*. in their study, where SLNs were detected in second compartment in 40% of patients.[23] It can be explained on the basis of multidirectional and complex lymphatic flow in the gastrointestinal tract. The incidence of metastasis was significantly higher in SLNs than in SLN-negative patients (76.92% vs. 33.34%) and was statistically significant (*P* < 0.005). Kitagawa *et al*. reported in their study that 27% of SLNs and only 2% of sentinel negative nodes had metastatic involvement in esophageal cancer.[22] The higher rate of lymph node metastases in SLN-negative patients could be explained by advanced stage of the disease in our patient population, leading to lymphatic blockade as explained above.

The SLN biopsy had an overall sensitivity of 90.90% and a false negative rate of 9% [Table 2]. The sensitivity of 90.9% in our study is higher than 71% reported by Hayashi *et al*.[18] using the radioisotope method; and 77% reported by Karube *et al*.[23] using the dye method but lower than that reported by other authors who had conducted sentinel lymph node biopsy in upper gastrointestinal cancers using dye or radioisotope methods.[19,21,22] The lower sensitivity of our procedure can be explained on the basis of higher tumor stage and metastases into the lymphatics causing blockage of lymphatics.[16,17] In the two patients in our study in whom lymph nodes failed to stain despite being visibly enlarged and presumably metastatic, was probably due to infiltration and blockade of lymphatics by the tumor cells and replacement of the lymph nodes with metastatic deposits. Therefore, the dye could not reach the lymph nodes leading to false negative results.

**Table 2:** Statistical values of the sentinel lymph nodes as per location and type of esophageal tumor

Statistical parameter (%)	Combined	Mid esophageal	Lower esophageal	Squamous cell carcinoma	Adenocarcinoma
Sensitivity	90.9	85.71	93.33	87.5	92.86
Accuracy	75.0	66.67	80.0	60.0	76.47
Positive predictive value	76.92	66.67	82.35	63.64	86.67
Negative predictive value	66.67	66.67	66.67	75.0	50.0

The accuracy of the SLN technique using methylene blue dye in predicting nodal metastases was 75%, which is comparable to the published data where radioisotope-labeled colloid and/or dye (isosulphan blue) were used to study esophageal and gastric cancers.[18,22,23] The negative predictive value and positive predictive value in our study were 66.67% and 76.92%, respectively [Table 2], which are slightly lower than the values published in the literature.[21,24,25]

The SLN technique is also feasible and safe, not only for laparoscopic but also for Robot-assisted laparoscopic lymph node dissection and esophageal anastomosis.[26] Merendino and Dillard’s procedure of reconstructing the esophagogastric passage by the interposition of an isoperistaltic segment of jejunum between the esophagus and stomach, is of utility only in localized lesions.[27] If the early neoplastic lesion is limited to the mucosa, endoscopic mucosal resection (EMR) could be considered because of the low chance of lymph node metastases. However, the technique of EMR has not yet been optimized, resulting in a high number of local cancer recurrences and a high need for endoscopic re-resections.[28] An adequately powered, prospectively randomized trial is needed to evaluate the benefits of SLN concept in this subset of patients.

We conclude that SLN mapping using methylene blue dye can be very helpful in predicting the metastatic involvement of the lymph nodes intraoperatively in esophageal malignancies, especially the lower esophageal cancers. Methylene blue is locally available, is not very costly and does not need any special equipment for its usage in the operation theater. Although more evidence from prospective, multicenter clinical trials is required, SLN mapping appears to be useful for individualizing multi-modal treatment for esophageal cancer. Application of sentinel node technology may in future allow limiting systematic lymphadenectomy to the rather small subgroup of patients who in fact have lymph node metastases.
